# Atypical vitelliform macular dystrophy misdiagnosed as chronic central serous chorioretinopathy: case reports

**DOI:** 10.1186/1471-2415-12-25

**Published:** 2012-07-20

**Authors:** Young Seob Lee, Eung-Suk Kim, Moosang Kim, Young-Gyun Kim, Hyung-Woo Kwak, Seung-Young Yu

**Affiliations:** 1 Department of Ophthalmology, School of Medicine, Kyung Hee University, Seoul, South Korea; 2 Department of Ophthalmology, School of Medicine, Eulji University, Daejeon, South Korea; 3 Department of Ophthalmology, School of Medicine, Kangwon national University, Chuncheon, South Korea

## Abstract

**Background:**

To report two cases of atypical vitelliform macular dystrophy misdiagnosed as chronic central serous chorioretinopathy.

**Case presentation:**

Two patients with incidentally discovered abnormalities of the retina without specific symptoms were referred to our hospital for consultation. Bilateral macula atrophic lesions were observed and optical coherence tomography revealed serous retinal detachment in the macula. Fluorescein angiography showed multiple leakages around the central hypofluorescent area and indocyanine green angiography showed partially dilated choroidal vessels. Fundus autofluorescence (FAF) showed a decreasing pattern of autofluorescence in the subretinal fluid area, and increasing autofluorescence at the border of the serous retinal detachment. Both patients were diagnosed with chronic central serous chorioretinopathy. Photodynamic therapy and intravitreal bevacizumab injection were administered for engorged choroidal vessels during follow-up, but neither patient showed improvement in symptoms or ophthalmologic findings. Based on re-evaluation by fundus photography, optical coherence tomography, fluorescein angiography, and comparison of the results of FAF with the first visit, vitelliform macular dystrophy was suspected and a definite diagnosis was made by electrooculography and genetic testing.

**Conclusion:**

In patients with continuous serous retinal detachment without response to photodynamic therapy or intravitreal bevacizumab injection, careful fundus exam and FAF can be used to diagnose atypical vitelliform macular dystrophy.

## Background

Vitelliform macular dystrophy (VMD) is an autosomal-dominant disease that can cause a gene mutation of bestrophin-1 (Best-1). This gene codes for a Ca2^+^-sensitive Cl^−^ channel protein located on the basolateral membrane of the retinal pigment epithelial cells [1-5]. There are several disease-causing mutations and the phenotypic appearance varies with the stage of the disease [6,7]. Previously, it was believed that congenital Best disease, adult-onset VMD, and autosomal dominant vitreoretinochoroidopathy were different disease entities. The identification of mutations in the Best1 gene, however, altered this notion and revealed that most individuals with these phenotypes had mutations in the same genes [8,9].

VMD can develop in various age groups, is usually bilateral, and induces a severe decrease of vision in the early stage, but in some cases visual acuity loss progresses insidiously. The typical fundus finings of VMD include a solitary, round or oval, slightly elevated, yellowish, subretinal lesion of the fovea [10,11]. Optical coherence tomography (OCT) shows thick hyper-reflective structures in the retinal pigmented epithelium (RPE) layer or between the RPE layer and photoreceptor layer, and serous retinal detachment [12]. Fluorescence angiography reveals early blockages and late intense hyperfluorescent circles with hypofluorescent central zones. Fundus autofluorescence (FAF) findings vary, but usually show hyperautofluorescence at the lesion site [13]. A decrease in the Arden ratio in the electrooculogram (EOG) is a pathognomic characteristic [14].

There is no specific treatment for VMD, but regular ophthalmologic examinations annually or twice annually to determine the occurrence of complications or comorbidities are necessary. Patients diagnosed with VMD should be frequently evaluated with an Amslers grid to elucidate changes or decreases in vision. As this is a genetic disorder, family members should also be examined. Some case reports indicate that photodynamic therapy and antivascular endothelial growth factor are effective against choroidal neovascularization, but to date there are no clinically established treatments available [15,16].

Here we report two cases of atypical VMD in patients who were misdiagnosed with chronic central serous chorioretinopathy (CSC) and did not respond to either photodynamic therapy or intravitreal bevacizumab injection.

## Case presentation

### Case 1

**Figure 1:**
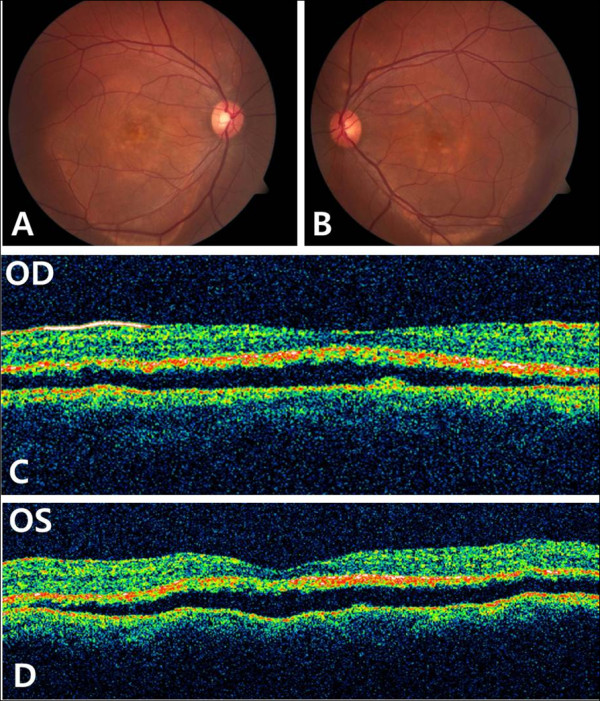
**Case 1. Fundus photographs and optical cohenrece tomography (OCT) at initial presentation. A**,**B**, Fundus photographs showed atrophic lesion in central area, both eyes. **C**,**D**, OCT findings of both eyes showed serous retinal detachment over a large area.

**Figure 2:**
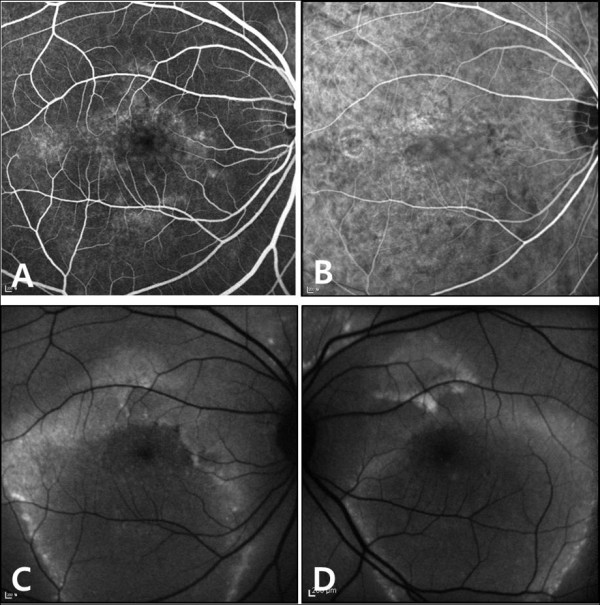
**A, Fluoresecein angiography findings of right eye revealed multiple leakages around the central hypofluorescence area. B**, Indocyanine green angiography showed dilated choroidal vessel. **C**,**D**, Fundus autofluorescence findings of both eyes revealed central hypoautofluorescence and ring-like hyperautofluorescence at margin of serous retinal detachment.

A 30-year-old man presented with incidentally discovered slightly decreased visual acuity in both eyes. He had no history of diabetes, hypertension, or any other medical history, and was not on any medications. The best-corrected visual acuity was 20/25 in the right eye and 20/22 in the left eye. Anterior segment examination showed no abnormal findings, but fundus examination and OCT revealed bilateral atrophic foveal lesions and extensive serous retinal detachment in the posterior pole of the retina (Figure 1). Fluorescein angiography showed bilateral hypofluorescence in the fovea, surrounding hyperfluorescence, and multiple fluorescent leakages. Indocyanine green angiography revealed slightly dilated choroidal vessels near the foveal region. FAF showed hypoautofluorescence in the central area and hyperautofluorescence at the border of the serous retinal detachment (Figure 2). Serologic examination showed no significant findings, and electroretinography revealed a normal waveform and amplitude in both eyes. The patient was diagnosed with chronic CSC, and at 10 months of follow-up, fundus examination and OCT showed the continued presence of subretinal fluid and no improvement in visual acuity. Photodynamic therapy was conducted in the areas of the hyperlucent choroidal vessels that were identified by indocyanine green angiography, but there was no improvement in symptoms or clinical presentation. At 15 months after the first visit, intravitreal antivascular endothelial growth factor was injected but again the patient showed no improvement. At 18 months, laser photocoagulation was applied for a suspected leaking point based on fluorescein angiography. Progressive decreases in visual acuity and serous retinal detachment continued through the long-term follow-up.

### Case 2

**Figure 3:**
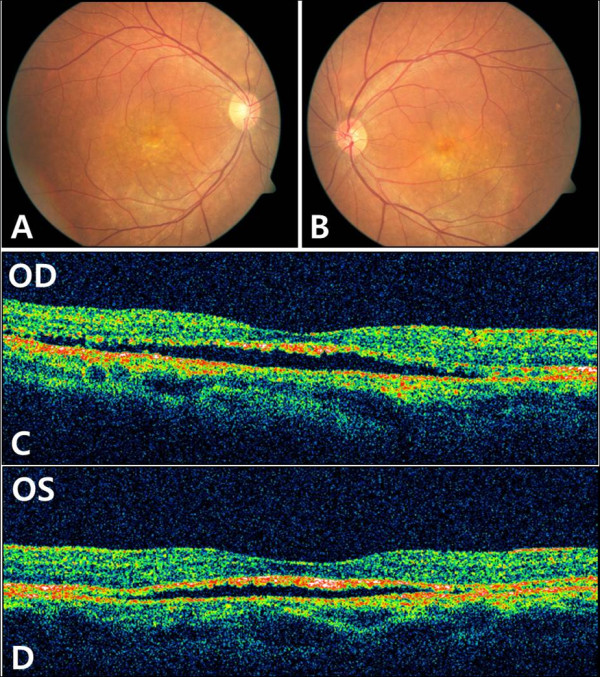
**Case 2 Case 2. A,B, Fundus photographs of both eyes showed atrophic lesion especillay near the macula, similar to case 1 patients. C**,**D**, OCT findings of both eyes also showed serous retinal detachment.

**Figure 4:**
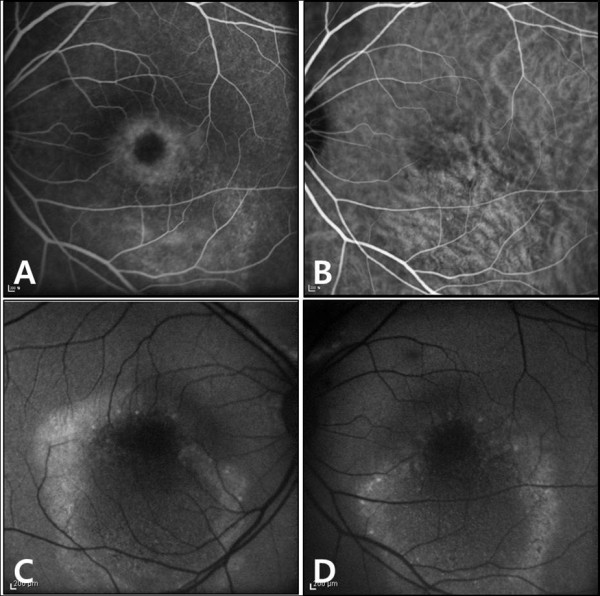
**A, Fluoresecein angiography findings of left eye revealed foveal hypofluorescene and surrounding hyperfluorescent ring. B**, Indocyanine green angiography showed moderated dilation of choroidal vessel. **C**,**D**, Fundus autofluorescence was increased at margin of serous retinal detachment, and general reduction of autofluorescent due to serous exudates in both eyes.

A 51-year-old man with no specific symptoms was referred to us for incidentally found macula abnormalities during a regular checkup. He had no diabetes, hypertension, underlying diseases, or family history. Bilateral best-corrected visual acuity was 20/22 with no clinical findings in the anterior segment, but fundus examination revealed atrophic lesions in the bilateral macula. Optical coherence tomography revealed a wide range of serous retinal detachments in both eyes (Figure 3). Fluorescein angiography showed both hypofluorescence of foveal lesions and ring-like hyperfluorescence in surrounding areas. Indocyanine green angiography showed engorgement of the choroidal vessels. FAF showed decreased autofluorescence in the central area and increased autofluorescence at the border of the serous retinal detachment (Figure 4). Bilateral chronic CSC was suspected and photodynamic therapy was applied during follow-up to the areas of dilated choroidal vessels based on indocyanine green angiography, but the serous retinal detachment did not improve.

**Figure 5:**
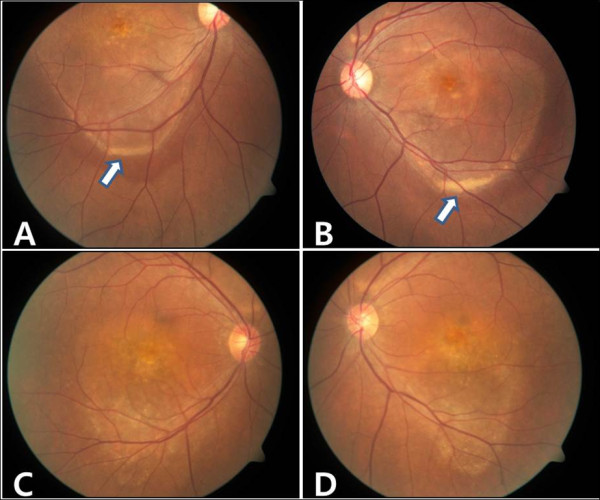
**A,B Case 1. Subretinal yellowish exudates with fluid level in the inferior part of serous retinal detachement are seen in photography of both eyes. (arrows) B,C Case 2.** There were little unclear, but yellowish exudates in inferior part of lesion were seen in both eyes.

**Figure 6:**
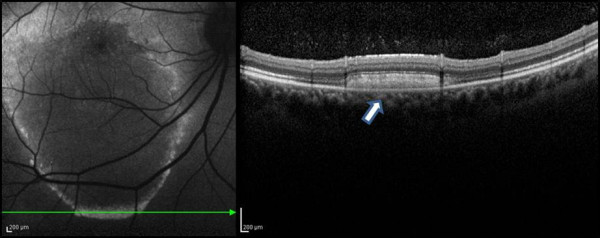
**Fundus autofluorescence (FAF) and spectral domain optical coherence tomography (SD-OCT) of case 1 patients.** The yellowish exudate lesion was seen hyperautofluorescent in FAF findings. SD-OCT revealed high reflectivity areas corresponding to subretinal exudates. (arrow).

**Figure 7:**
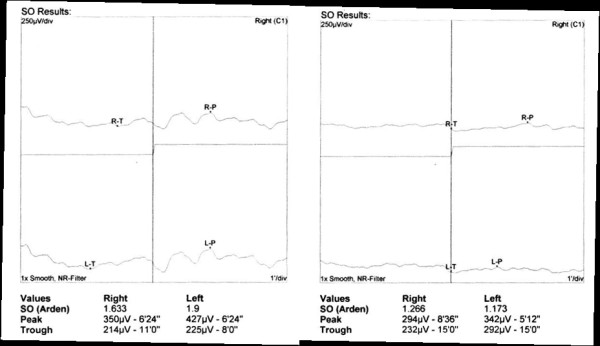
The electro-oculograms were abnormal with an Arden ratio of 1.633, 1.9 (left, case 1) and 1.266, 1.173 (right, case 2) in both eyes, respectively.

These two cases of suspected chronic CSC showed continuous serous retinal detachment even after the photodynamic therapy over 3 years, multiple leakages in fluorescein angiography, and hyperlucent choroidal vessels in indocyanine green angiography. The records of these two cases were therefore reviewed in more detail. Yellowish deposits were observed at the border of the serous retinal detachment areas (Figure 5), and OCT revealed hyper-reflective lesions between the RPE layer and outer layer of the retina. FAF revealed ring-like hyperautofluorescence around the serous retinal detachment, which is rarely seen in chronic CSC (Figure 6). This clinical appearance is seen in atypical VMD and based on the EOGs of these patients, the first patient had a decreased Arden ratio of 1.633 OD, 1.9 OS, and the second patient had 1.266 OD and 1.173 OS (Figure 7). For definite diagnosis, genetic examination was conducted and both patients were VMD2 gene-positive, and thus the diagnosis of VMD was confirmed.

## Discussion

VMD is a rare disease with autosomal dominant inheritance. It can occur in any age group, but when it is detected in younger patients between 3 and 15 years of age, it is considered to be a Best vitelliform macular dystrophy, and in patients ages between 30 and 50 years, it is considered to be adult-onset VMD with differences in the clinical appearance. It was recently discovered, however, that both are due to abnormalities of the Best1 gene with different phenotypes and its primary cause is abnormalities in bestrophin function [8,9].

Typical fundus findings in VMD are egg yolk-like, round or oval, lesions found in the bilateral macula. The exact location of the deposits in the retina is unclear, but based on spectral domain-OCT [17], they are located beneath the sensory retina, causing changes in the photoreceptor inner segment/outer segments. The cause of the egg yolk-like yellowish deposits is unknown, but may be due to unphagocytosed outer segments that accumulate because of the lack of apposition of the outer membrane segments to the RPE. The persistence of the material in the subretinal space increases the likelihood of the formation of lipofuscin precursors, which are autofluorescent and susceptible to oxidative damage [12]. In histologic examination, these lipofuscins appear as egg yolk-like lesions [18]. In the advanced disease state, lipofuscins normally get reabsorbed or disappear, and cause atrophy in the RPE. The findings of fluorescein angiography vary based on the yellowish lesions, usually hypofluorescence in the central lesions and a window defect can be seen, and in FAF, the fluorescence of the lesions is irregularly increased or decreased [19].

In EOG, a decrease in the Arden ratio is a distinctive feature of VMD [14], due to a defect in Ca^2+^ activated Cl- channels of the RPE basolateral membrane that expresses bestrophin. Until recently, EOG has been the most accurate test for determining the cause of the disease as well as genetic examination.

At a young age, bilateral neurosensory retinal detachment can be caused by infections, inflammation, collagen-vascular disease, tumor, age-related macular degeneration, polypoidal choroidal vasculopathy, and CSC. In the presented cases, there were no symptoms of infection or inflammation, and both systemic disease and tumor were also excluded. Age-related macular degeneration usually occurs at an advanced age, and polypoidal choroidal vasculopathy can occur at a young age, but polypoidal dilation was not observed on indocyanine green angiography so it was excluded. Therefore, chronic CSC was suspected based on the examinations and clinical features in these cases.

Based on the clinical features, the patients were diagnosed with chronic CSC and received photodynamic treatment for months. Case 1 received intravitreal bevacizumab injection and laser photocoagulation therapy to coagulate the leaking points but serous retinal detachment persisted for 2 years. Also, there was no change in the autofluorescence for several years compared to the baseline.

**Figure 8:**
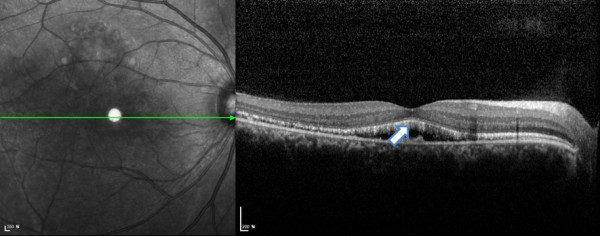
The photoreceptor inner segment/outer segment junction was intact (arrow) which was confirmed by SD-OCT, even with RPE atrophy after long standing serous retinal detachment.

The common clinical features of the two cases led us to review the patient records since the first visit. Although both cases had a prolonged disease state, there was no decrease in visual acuity, atrophic lesions in the macula region were observed, fluorescein angiography showed hypofluorescence in the macula, hyperfluorescence in the surrounding areas, and a window defect in the surrounding region due to atrophy in the RPE layer. Especially in FAF, both cases showed increased autofluorescence in the border of serous retinal detachment, and yellowish lesions were located below and around the serous retinal detachment in the fundus photograph. Spectral domain-OCT was used to scan the area and showed hyper-reflective lesions between the RPE layer and the photoreceptor IS/OS junction. We suspected VMD in these two cases, because when VMD progresses, the yellowish egg yolk-like lesions in VMD get absorbed spontaneously or form fluid, which can make it difficult to find definite lesions, and we observed atrophic lesions of the macula and continuous subretinal fluid. The source of the subretinal fluid appears to be related to the underlying defect in VMD2, which involves bestrophin, a Ca^2+^ -sensitive Cl^–^ channel protein found in the basolateral portion of the RPE cells [12]. The EOG revealed a decrease in the Arden ratio in both patients. Additionally, genetic examination revealed the mutation in VMD2 gene, which led to a definite diagnosis. The reasons why visual acuity was maintained even after long-lasting VMD as follows were that the patient was comparably young, and photoreceptor inner segment/outer segment junction was not disrupted, which was confirmed by SD-OCT, even with RPE atrophy after long standing serous retinal detachment (Figure 8).

In the case of CSC, as the disease state progresses hyperautofluorescence or hypoautofluorescence can be seen in FAF, but it is uncommon to find an overall decrease in autofluorescence in the center and an increase in autofluorescence at the border of the serous retinal detachment in CSC. Normally, egg yolk-like lesions in VMD are lipofuscins, which can lead to an increase in autofluorescence in the lesion areas. In these cases, the autofluorescence decreased due to the absorption of lesions in the central area and continuous serous retinal detachment, and autofluorescence increased due to the existence of deposits at the bottom border of the serous retinal detachment and around the area of the serous detachment. Especially in the second case, fundus photography and slit lamp examination revealed no yellowish deposits, but FAF showed hyperautofluorescence in the border of serous retinal detachment, so it was very helpful for making a diagnosis.

## Conclusions

In patients who do not respond to treatment for continuous bilateral serous retinal detachment, it is necessary to differentiate atypical cases of VMD with careful ophthalmologic examination. FAF may be helpful for diagnosis.

## Consent

Written informed consent was obtained from the patient for publication of this case report and any accompanying images. A copy of the written consent is available for review by the Editor in-Chief of this journal.

## Competing interests

The authors declare that they have no competing interests.

## Authors’ contribution

YSL, ESK, MSK, YGK, HWK and SYY treated the patient and in doing so acquired the case data; they were also involved with drafting of the manuscript. All authors read and approved the final manuscript.

## Pre-publication history

The pre-publication history for this paper can be accessed here:

http://www.biomedcentral.com/1471-2415/12/25/prepub

## References

[B1] Forsman K Graff C Nordström S Johansson K Westermark E Lundgren E The gene for Best’s macular dystrophy is located at 11q13 in a Swedish family Clin Genet 1992 42 156 159 1395087 10.1111/j.1399-0004.1992.tb03229.x

[B2] Stone EM Nichols BE Streb LM Kimura AE Sheffield VC Genetic linkage of vitelliform macular degeneration (Best’s disease) to chromosome 11q13 Nat Genet 1992 1 246 250 10.1038/ng0792-246 1302019

[B3] Petrukhin K Koisti MJ Bakall B Li W Xie G Marknell T Identification of the gene responsible for Best dystrophy Nat Genet 1998 19 241 247 10.1038/915 9662395

[B4] Marmorstein AD Marmorstein LY Rayborn M Wang X Hollyfield JG Petrukhin K Bestrophin, the product of the Best vitelliform macular dystrophy gene (VMD2), localizes to the basolateral plasma membrane of the retinal pigment epithelium Proc Natl Acad Sci U S A 2000 97 12758 12763 10.1073/pnas.220402097 11050159 PMC18837

[B5] Sun H Tsunenari T Yau KW Nathans J The vitelliform macular dystrophy protein defines a new family of chloride channels Proc Natl Acad Sci U S A 2002 99 4008 4013 10.1073/pnas.052692999 11904445 PMC122639

[B6] Gass JDM Stereoscopic Atlas of Macular Disease: Diagnosis and Treatment 1997 4 St. Louis: Mosby 304 311

[B7] Deutman AF Hoyng CB Ryan SJ, Ogden TE, Hinton DR, Schachat AP Macular dystrophies 2001 3 St. Louis: Mosby 1210 1257

[B8] Boon CJ Klevering BJ Leroy BP Hoyng CB Keunen JE den Hollander AI The spectrum of ocular phenotypes caused by mutations in the BEST1 gene Prog Retin Eye Res 2009 28 187 205 10.1016/j.preteyeres.2009.04.002 19375515

[B9] Booij JC Boon CJ van Schooneveld MJ ten Brink JB Bakker A de Jong PT Course of visual decline in relation to the Best1 genotype in vitelliform macular dystrophy Ophthalmology 2010 117 1415 1422 10.1016/j.ophtha.2009.11.044 20381869

[B10] Gass JDM A clicopathologic study of a peculiar foveomacular dystrophy Trans Am Ophthalmol Soc 1974 72 139 156 4142662 PMC1311393

[B11] Lim JI Enger C Fine SL Foveomacular dystrophy Am J Ophthalmol 1994 117 1 6 8291575 10.1016/s0002-9394(14)73007-7

[B12] Spaide RF Noble K Morgan A Freund KB Vitelliform macular dystrophy Ophthalmology 2006 113 1392 1400 10.1016/j.ophtha.2006.03.023 16877078

[B13] Chung JE Spaide RF Fundus autofluorescence and vitelliform macular dystrophy Arch Ophthalmol 2004 122 1078 1079 10.1001/archopht.122.7.1078 15249383

[B14] Deutman AF Electro-oculography in families with vitelliform dystrophy of the fovea. Detection of the carrier state Arch Ophthalmol 1969 81 305 316 10.1001/archopht.1969.00990010307001 5774285

[B15] Andrade RF Farah ME Costa RA Photodynamic therapy with verteporfin for subfoveal choroidal neovascularization in best disease Am J Ophthalmol 2003 136 1179 1181 10.1016/S0002-9394(03)00711-6 14644242

[B16] Leu J Schrage NF Degenring RF Choroidal neovascularization secondary to Best’s disease in a 13-year-old boy treated by intravitreal bevacizumab Graefes Arch Clin Exp Ophthalmol 2007 245 1723 1725 10.1007/s00417-007-0604-7 17605026

[B17] Puche N Querques G Benhamou N Tick S Mimoun G Martinelli D High-resolution spectral domain optical coherence tomography features in adult onset foveomacular vitelliform dystrophy Br J Ophthalmol 2010 94 1190 1196 10.1136/bjo.2009.175075 20576764

[B18] O’Gorman S Flaherty WA Fishman GA Berson EL Histopathologic findings in Best’s vitelliform macular dystrophy Arch Ophthalmol 1988 106 1261 1268 10.1001/archopht.1988.01060140421045 3415551

[B19] Renner AB Tillack H Kraus H Kohl S Wissinger B Mohr N Morphologic and functional charateristics in adult vitelliform macular dystrophy Retina 2004 24 929 939 10.1097/00006982-200412000-00014 15579992

